# Blood pressure, frailty status, and all-cause mortality in elderly hypertensives; The Nambu Cohort Study

**DOI:** 10.1038/s41440-021-00769-0

**Published:** 2021-10-15

**Authors:** Taku Inoue, Mitsuteru Matsuoka, Tetsuji Shinjo, Masahiro Tamashiro, Kageyuki Oba, Masanori Kakazu, Takuhiro Moromizato, Osamu Arasaki, Hisatomi Arima

**Affiliations:** 1 Cardiovascular Medicine, Tomishiro Central Hospital, Tomigusuku, Japan; 2 Department of Preventive Medicine and Public Health, Faculty of Medicine, Fukuoka University, Fukuoka, Japan; 3 Matsuoka Clinic, Tomigusuku, Japan; 4 Cardiovascular Medicine, Yuai Medical Center, Tomigusuku, Japan; 5 Renal and Rheumatorogy Division, Nanbu Medical Center and Children’s Medical Center, Haebaru, Japan

**Keywords:** Frailty, Blood pressure, Mortality

## Abstract

Antihypertensive therapy is pivotal for reducing cardiovascular events. The 2019 Guidelines for the Management of Hypertension set a target blood pressure (BP) of <140/90 mmHg for persons older than 75 years of age. Optimal BP levels for older persons with frailty, however, are controversial because evidence for the relationship between BP level and prognosis by frailty status is limited. Here, we evaluated the relationship between systolic BP and frailty status with all-cause mortality in ambulatory older hypertensive patients using data from the Nambu Cohort study. A total of 535 patients (age 78 [70–84] years, 51% men, 37% with frailty) were prospectively followed for a mean duration of 41 (34–43) months. During the follow-up period, 49 patients died. Mortality rates stratified by systolic BP and frailty status were lowest in patients with systolic BP < 140 mmHg and non-frailty, followed by those with systolic BP ≥ 140 mmHg and non-frailty. Patients with frailty had the highest mortality regardless of the BP level. The adjusted hazard ratios (95% confidence intervals) of each category for all-cause mortality were as follows: ≥140 mmHg/Non-frailty 3.19 (1.12–11.40), <140 mmHg/Frailty 4.72 (1.67–16.90), and ≥140 mmHg/Frailty 3.56 (1.16–13.40) compared with <140 mmHg/Non-frailty as a reference. These results indicated that frail patients have a poor prognosis regardless of their BP levels. Non-frail patients, however, with systolic BP levels <140 mmHg had a better prognosis. Frailty may be a marker to differentiate patients who are likely to gain benefit from antihypertensive medication among older hypertensives.

## Introduction

Hypertension is a significant risk factor for cerebrovascular disease [1–3], and antihypertensive therapy can reduce the onset, progression, and recurrence of cerebrovascular disease leading to the risk of deterioration of quality of life or death [4–6]. There is a log-linear association between blood pressure (BP) levels and cardiovascular mortality hazard ratios (HRs), but the slope is smaller in older individuals, and the contribution of BP to the development of cerebrovascular events is lower [1, 7]. When this study started in 2017, elderly hypertensive patients were managed according to 2014 Japanese Society of Hypertension Guidelines for the Management of Hypertension (JSH 2014) [8], which recommended BP control of <140/90 for those aged 65–74 years and <150/90 mmHg for those aged 75 years and older. Based on the results of randomized control trials of antihypertensive treatment for the elderly, the 2019 Japanese Society of Hypertension Guidelines for the Management of Hypertension (JSH 2019) set the target BP value for antihypertensive therapy in persons 75 years of age or older as <140/90 mmHg [9]. Frailty should be considered as a factor in the debate on optimal BP levels for older hypertensive patients. Frailty is a state of increased vulnerability resulting from an age-associated decline in reserve and function over multiple physiologic systems such that the ability to cope with everyday or acute stressors is compromised [10].

Although frailty is associated with impaired activities of daily living due to deterioration in physical function, the biggest problem with frailty is increased hospitalization and worsening life expectancy [11]. Furthermore, while patients with frailty have a higher prevalence of comorbidities [12], cardiovascular risk levels are somewhat lower [12–14], and lower BP levels in frailty are associated with poor prognosis [15]. Due to the limited evidence available on antihypertensive therapy in patients with frailty [16, 17], coupled with the absence of clear diagnostic criteria for frailty [17], there is no consensus on the optimal BP level for hypertensive patients with frailty, which currently relies on the clinician’s experience. In this background, we examined how frailty status affects initial BP levels and subsequent mortality rate in older hypertensive patients using data from the Nambu Cohort Study, a prospective observational study of older outpatients.

## Methods

### Study patients and data collection

**Fig. 1:**
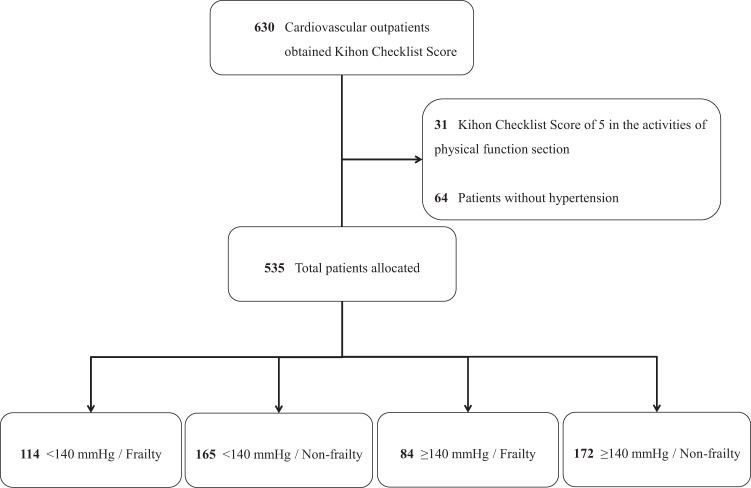
Diagram of the study

The Nambu Cohort Study is a prospective cohort study of older patients in an outpatient setting in the southern area of Okinawa, Japan, that began in 2017 [13]. We collect and accumulate data on the anthropometric indices, comorbidities, cardiovascular risk profiles, blood test data, prescribed drugs, and physical function of outpatients aged 65 years or over. Our ultimate goal is to provide evidence for factors contributing not only to a patients’ disease prognosis, but also those contributing to a better healthy life expectancy. Patients who could complete the interview conducted by the medical assistant to provide information regarding frailty were continuously registered. Among the original registry, those with a Kihon Checklist (KCL) score of 5 (the worst) in the physical function section, and those without diagnosed hypertension were excluded. Finally, 535 patients were allocated to four categories according to their initial BP and frailty status as follows; <140 mmHg/Frailty (*n* = 114), <140 mmHg/Non-frailty (*n* = 165), ≥140 mmHg/Frailty (*n* = 84), and ≥140 mmHg/Non-frailty (*n* = 172) (Fig. 1).

### Definition of clinical disease

A trained nurse measured the patient’s height and body weight before the outpatient examination. Body weight and height were measured with the patient in indoor clothing without shoes. Body mass index was calculated as the weight in kilograms divided by the square of the height in meters (kg/m^2^). Blood pressure (BP) was measured once in the sitting using an automatic BP monitor (HBP-9020, Omron Corp., Kyoto, Japan) after resting for about 10 min on either the left or right upper arm. All blood samples were obtained from the antecubital vein after overnight fasting. Enzymatic methods were used to measure serum creatinine, total cholesterol, triglycerides, and low-density lipoprotein cholesterol levels, and the direct method was used to measure high-density lipoprotein cholesterol. The hexokinase/glucose-6-phosphate dehydrogenase method was used to measure fasting plasma glucose. High-performance liquid chromatography was used to measure the hemoglobin A1c level. Blood cell measurements such as leukocyte count, hematocrit, hemoglobin level, and platelet count were quantified using an automated blood cell counter. A Smedley-type (mechanical) hand grip dynamometer (TTM, Tokyo, Japan) was used to measure grip strength.

In this study, the KCL was used to diagnose frailty. The KCL was developed by the Japanese Ministry of Health, Labor and Welfare to identify elderly individuals at risk of requiring care/support [18]. The KCL is a simple self-reporting yes/no survey comprising 25 questions regarding instrumental and social activities of daily living, physical functions, nutritional status, oral function, cognitive function, and depressive mood. Satake et al. verified the clinical usefulness of the KCL as a frailty indicator [19]. Patients with a KCL Score of 0–3 were diagnosed as robust, 4–7 with pre-frailty, and 8 or over with frailty [19]. Well-trained medical staff asked the patients about the content of the KCL before the medical examination and recorded the patients’ responses.

### Statistical analysis and ethics

Continuous variables are expressed as median (interquartile range) and categorical variables as percentages. The median values of continuous variables were compared using the Kruskal–Wallis test. Categorical variables were compared using the chi-square test. The Kolmogorov–Smirnov test was used to evaluate the distribution normality. The mortality rate was calculated as the total number of outcomes during the follow-up period divided by 100 patient-years. For statistical analysis, the patients were stratified into two groups according to their frailty status, with robust and pre-frail being considered non-frail. A multivariate analysis was performed using the Cox proportional hazard model to evaluate the relationship of BP and frailty status with all-cause mortality. Hazard ratios (HRs) and 95% confidence intervals (CIs) were calculated and adjusted for age, sex, diabetes, dyslipidemia, obesity, chronic kidney disease, stroke, coronary artery disease, heart failure, and antihypertensive drug use such as calcium channel blockers and renin-angiotensin system inhibitors. Interaction between systolic BP and frailty was investigated by adding interaction terms to the statistical models. Statistical analyses performed using JMP 9.0.2 (SAS Institute Inc., Cary, NC, USA). All statistical tests were two-sided, and *p* < 0.05 was considered statistically significant. The study design was approved by the local ethics committee (Social Medical Corporation Yuaikai, Okinawa, Japan H30R009) and conducted according to the Declaration of Helsinki.

## Results

**Fig. 2:**
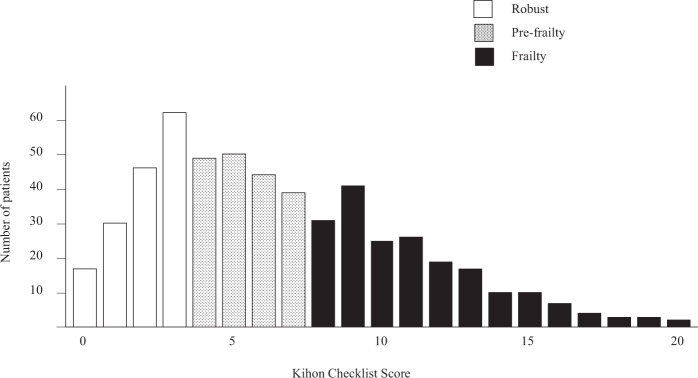
Distribution of the Kihon Checklist score of 535 hypertensive patients in the Nambu Cohort Study

A total of 535 older hypertensive patients (age 78 [71–84] years, 51% men, 37% with frailty) were prospectively followed for a mean duration of 41 (34–43) months. The prevalence of robustness, pre-frailty, and frailty was 29%, 34%, and 37%, respectively (Fig. 2). Table 1 shows the baseline characteristics of the patients stratified by systolic BP and frailty status. Within the same systolic BP category, frail patients were older with a higher prevalence of female sex, heart failure, stroke, peripheral arterial disease, and atrial fibrillation, than patients without frailty. Furthermore, patients with frailty had lower BP, body mass index, hand grip strength, estimated glomerular filtration rate, serum lipids, hemoglobin, and hematocrit levels than those without frailty, while uric acid, blood glucose, and HbA1c levels, and white blood cell count were similar (Table 2). During the median follow-up period of 41 (34–43) months, 49 patients died.

**Table 1:** Baseline characteristics of 535 patients in the Nambu Cohort Study stratified by blood pressure and frailty status

	<140 mmHg		≥140 mmHg		*p*
	Frailty *n* = 114	Non-frailty *n* = 165	Frailty *n* = 84	Non-frailty *n* = 172	
Age (years)	82 (75–87)	75 (69–80)	84 (80–89)	76 (68–81)	<0.0001
Male (%)	44	58	30	60	<0.0001
Comorbidity at entry					
Diabetes (%)	32	30	29	29	0.9613
Dyslipidemia (%)	57	67	68	65	0.2859
Obesity (%)	40	50	48	56	0.0655
Coronary artery disease (%)	25	22	24	15	0.1762
Chronic heart failure (%)	32	16	26	9	<0.0001
Stroke (%)	42	28	37	17	<0.0001
Peripheral arterial disease (%)	12	4	14	5	0.0031
Atrial fibrillation (%)	26	13	15	11	0.0033
Chronic kidney disease (%)	52	34	57	34	0.0002
Antihypertensive medications					
ACE-I or ARB (%)	61	56	49	58	0.3464
Calcium channel blocker (%)	66	73	73	77	0.5952

**Table 2:** Baseline anthropometric and laboratory findings of 535 patients in the Nambu Cohort Study stratified by baseline blood pressure and frailty status

	<140 mmHg		≥140 mmHg		
	Frailty *n* = 114	Non-frailty *n* = 165	Frailty *n* = 84	Non-frailty *n* = 172	*p*
Systolic blood pressure (mmHg)	126 (118–134)	129 (120–135)	152 (145–160)	150 (143–158)	<0.0001
Diastolic blood pressure (mmHg)	64 (56–72)	68 (62–75)	72 (66–78)	78 (72–85)	<0.0001
Heart rate (bpm)	75 (66–82)	73 (63–85)	76 (64–84)	77 (67–87)	0.02171
Body mass index (kg/m^2^)	24.0 (21.8–27.0)	24.9 (22.4–27.2)	24.9 (21.8–27.0)	25.8 (23.7–28.4)	0.0281
Hand grip strength (kg) men	25.0 (19.8–29.3)	29 (24.6–34.0)	21.0 (15.5–28.0)	29.0 (26.0–34.0)	<0.0001
women	13.0 (11.5–15.5)	19.5 (15.0–24.0)	13.0 (9.0–16.0)	20.0 (16.5–23.0)	<0.0001
eGFR (ml/min/1.73 m^2^)	59 (46–71)	65 (55–75)	55 (42–67)	67 (55–80)	<0.0001
Uric acid (mg/dl)	5.5 (4.7–6.7)	5.8 (4.9–6.5)	5.6 (4.6–6.6)	5.7 (4.8–6.7)	0.9152
Fasting blood sugar (mg/dl)	111 (100–138)	109 (101–129)	111 (99–133)	110 (101–125)	0.9349
HbA1c (%)	5.8 (5.5–6.1)	5.9 (5.7–6.2)	5.8 (5.5–6.2)	5.9 (5.6–6.2)	0.3391
Total cholesterol (mg/dl)	176 (154–195)	180 (165–202)	175 (154–196)	190 (169–210)	0.0001
HDL cholesterol (mg/dl)	51 (43–61)	58 (46–66)	53 (42–65)	56 (48–65)	0.0317
Triglycerides (mg/dl)	101 (76–141)	99 (76–132)	107 (81–139)	107 (81–139)	0.2624
LDL cholesterol (mg/dl)	94 (80–119)	99 (84–120)	95 (81–115)	106 (90–124)	0.0151
White blood cell (/mm^3^)	59 (49–71)	69 (50–74)	61 (51–75)	64 (54–77)	0.0983
Hemoglobin (g/dl)	12.3 (10.9–13.9)	13.1 (12.5–14.2)	12.2 (11.3–13.4)	13.5 (12.6–14.5)	<0.0001
Hematocrit (%)	38 (34–42)	40 (38–43)	38 (35–41)	42 (39–44)	<0.0001

*eGFR* estimated glomerular filtration rate, *HDL* high-density lipoprotein, *LDL* low-density lipoprotein

**Fig. 3:**
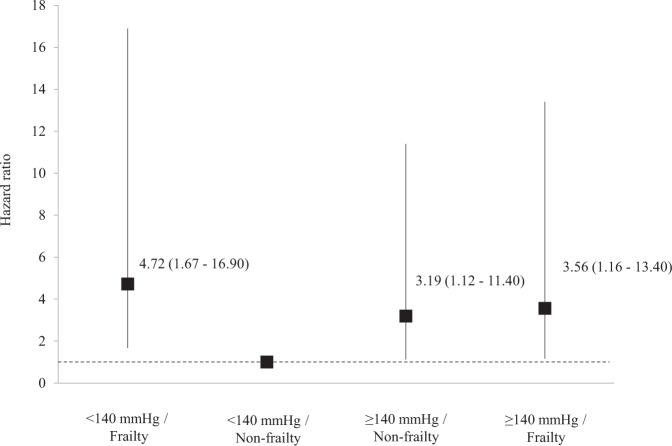
Adjusted hazard ratios and 95% confidence intervals for all-cause mortality for the combination of blood pressure and frailty status of 535 hypertensive patients in the Nambu Cohort Study

The adjusted HRs and 95% CIs for all-cause mortality of each systolic BP and frailty status category are shown in Fig. 3. Compared with patients in the <140 mmHg/Non-frailty category as a reference, patients with frailty had a higher HR for all-cause death regardless of their BP levels (<140 mmHg/Frailty; HR 4.72 [95% CI 1.67–16.90], ≥140 mmHg/Frailty; HR 3.56 [95% CI 1.16–13.40]), and ≥140 mmHg/Non-frailty; HR 3.19 [95% CI 1.12–11.40]; Fig. 3). We again performed a multivariate analysis with systolic BP and frailty as independent covariates to assess whether systolic BP and frailty were independently associated with all-cause mortality. The results showed that frailty doubled the risk of all-cause mortality compared to non-frail (HR 2.00, 95% CI 1.01–4.05, *p* = 0.0467). On the other hand, systolic BP ≥ 140 mmHg did not significantly increase the risk of all-cause mortality compared with <systolic BP140 mmHg (HR 1.17, 95% CI 0.65–2.11, *p* = 0.6045).

The number of all-cause deaths and mortality rates for each systolic BP level and frailty status is shown in Table 3. The mortality rate was lowest in patients with a systolic BP < 140 mmHg/Non-frailty, followed by those with systolic BP ≥ 140 mmHg/Non-frailty. The mortality rate was higher in patients with frailty than in those without frailty. In addition, frail patients with lower systolic BP level had higher mortality rate than those with higher systolic BP level (Table 3).

**Table 3:** Number of deaths, mortality rates per 100 patients year and hazard ratios for all-cause death by blood pressure and frailty status of 535 hypertensive patients in the Nambu Cohort Study

	Systolic blood pressure level	
	<140 mmHg	≥140 mmHg
Non-frailty		
N	165	172
Median age	75 (69–80)	76 (68–81)
All-cause death	4	13
Mortality rate (/100 patients year)	0.73	2.36
Hazard ratios (95% confidence intervals)^a^	Reference	2.99 (1.06–10.63)
Frailty		
N	114	84
Median age	82 (75–87)	84 (80–89)
All-cause death	18	14
Mortality rate (/100 patients year)	5.63	5.46
Hazard ratios (95% confidence intervals)^a^	5.18 (1.84–18.44)	3.78 (1.24–14.15)

^a^Adjusted for age and sex

Re-evaluation of those patients aged 75 years or older revealed that HRs increased and 95% CIs varied more, but the trend was the same as for all patients (Data are not shown).The marginal significance of an interaction between systolic BP and frailty partly supports these results (*P* = 0.085 for interaction).

## Discussion

**Fig. 4:**
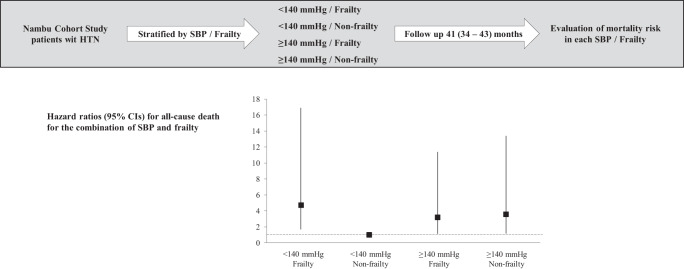
Graphical Abstract: In elderly hypertensives, higher blood pressure levels are associated with an increased risk of death in non-frailty, while frailty has a greater mortality risk regardless of blood pressure levels

The results of the present study in which older hypertensive patients were followed in an outpatient setting demonstrated that compared to non-frail patients with systolic BP levels <140 mmHg, frail patients with systolic BP levels <140 mmHg had highest risk of all-cause mortality, followed by systolic BP levels ≥140 mmHg. In non-frail patients, the risk of all-cause mortality was approximately 3.2 times higher in patients with systolic BP levels ≥140 mmHg than in those with systolic BP levels <140 mmHg, and the trend was similar even when subjects were restricted to those aged 75 years or older (Fig. 4).

### Background of aging and frailty

Adults 75 years or over undergo many age-related physiologic and pathologic changes, such as BP regulation abnormalities and are likely to have complications such as frailty, cognitive dysfunction, and polypharmacy [20]. Therefore, therapeutic strategies for the elderly should be different from those for the young and middle-aged. Because older adults are a heterogeneous group, uniformly classifying them according to their age should be avoided. Frailty is considered a state of impaired homeostasis in response to stress and accelerated biologic aging [21]. One of the mechanisms contributing to the progression of frailty is inflammaging, a low-grade chronic inflammation associated with aging. Inflammatory cytokines activate muscle degradation to produce amino acids for energy or cleave antigenic peptides [22], which leads to hemodynamic instability. Studies evaluating the association between aging frailty and cardiovascular risk factors indicate that BP, lipids, and body weight levels are reduced in frail individuals, although comorbidities are more prevalent [12–14]. Thus, in frail individuals, cardiovascular risk factors accumulate, while the level of each cardiovascular risk factor is somewhat reduced. In prescribing antihypertensive therapy for older patients, these characteristics of frailty should be kept in mind.

### Interventional and epidemiologic studies of older hypertensive patients

The results of a randomized controlled study on aggressive antihypertensive therapy targeting a systolic BP < 140 mmHg in patients aged 75 years or older showed that antihypertensive therapy targeting a systolic BP of <140 mmHg did not reduce the number of combined cerebrovascular events compared with therapy targeting a systolic BP ≥ 140 mmHg, but did reduce all-cause and cerebrovascular disease mortality [23–28]. On the basis of these results, the JSH2019 set an antihypertensive therapy target for hypertensive patients aged 75 years or older at a systolic BP < 140 mmHg [9]. On the other hand, antihypertensive treatment of hypertensive patients with frailty has been discussed. Randomized controlled studies examining the antihypertensive treatment of frail patients include the HYpertension in the Very Elderly Trial (HYVET) [17] and the Systolic Blood Pressure Intervention Trial (SPRINT) [16], in which the authors concluded that there is no need to change the antihypertensive goal for patients with frailty. The interpretation of their results, however, has been questioned. First, although frailty is associated with lower levels of cardiovascular risk factors [12–14], the patients in both studies had higher levels of cardiovascular risk factors. Also, patients with comorbidities often found in frailty were excluded in these studies. Thus, many of the patients judged to be frail in the two studies could be classified as robust-prefrail by other criteria [29]. Furthermore, the BP values in SPRINT are not measured in a typical office setting. Extrapolation of results obtained in such a particular group of patients to the real world is not feasible.

In an epidemiologic study evaluating systolic BP and life expectancy in older hypertensive patients, low systolic BP levels increased total mortality [15]. In contrast, high systolic BP levels increased the risk of cardiovascular mortality, and there was a U-shaped association between systolic BP and the total mortality risk [30–32]. The optimal systolic BP level with the lowest risk of death was 130–150 mmHg [30, 31], and the association between systolic BP level and life expectancy was similar in the older general population [33, 34]. The association between systolic BP and frailty or dementia has also been reversed by background factors such as age, antihypertensive treatment, or comorbidities [35]. Therefore, the assessment of the association between BP levels and prognosis in older people should carefully be interpreted, taking into account a variety of factors.

The results of the present Nambu Cohort study, reconsidered in conjunction with existing evidence, led to the following findings. First, frail patients have lower BP levels. Second, frail patients have a higher risk of all-cause mortality regardless of BP level. Third, non-frail patients have a lower risk of all-cause mortality with lower BP levels. The absence of an interaction between systolic BP and frailty supports these results. The U-shaped relationship between systolic BP level and all-cause mortality shown in epidemiologic studies of older hypertensive patients can be explained by the combination of frailty and systolic BP levels.

### Future perspectives

The JSH2019 set the antihypertensive therapy target for ambulatory hypertensive patients at <140/90 mmHg, with frailty to be determined individually [9]. Randomized controlled trials in older patients, especially those evaluating frailty, are limited, and there are many challenges in generalizing their results. Individually assessed indications of potential benefits and harms of antihypertensive therapy rely mainly on the clinician’s experience. Our results are consistent with the JSH2019 recommendations and suggest that an assessment of frailty has an essential role in determining the treatment strategy for older hypertensive patients. While antihypertensive therapy aimed at <140 mmHg may be effective in older non-frail patients, weaning a patient from frailty should be prioritized over therapy targeting BP levels in frail patients. Frailty may be an important marker to distinguish whether antihypertensive therapy aiming for systolic BP < 140 mmHg can reduce all-cause mortality in older hypertensive patients.

### Limitation

The present study has several limitations. First, the results of this study were derived from a prospective cohort analysis. Due to its observational nature, we may not have been able to rule out the effects of confounding factors, and the results show only an association and not causation. Second, the patients in this study were outpatients of a hospital or clinic, most of whom have multiple comorbidities. Therefore it is undeniable that discrepancies exist in the interpretation of the results with the general population or nursing home residents. Third, because β-blockers and diuretics are often administered for heart failure, only calcium channel blockers, angiotensin-converting enzyme inhibitors, and angiotensin receptor blockers were evaluated as antihypertensive agents. Fourth, BP values were evaluated using only the BP obtained at the initial office visit, but BP values during the follow-up were not considered. Lastly, the patients were residents of a relatively limited area—the southern part of Okinawa, Japan. Although the present analysis results were consistent with previous findings, these issues should be considered when interpreting the results of the present study.

## Conclusion

The results of this prospective cohort study of ambulatory hypertensive patients indicated that frail patients had a higher risk of all-cause mortality than non-frail patients, regardless of BP level. In contrast, in non-frail patients, systolic BP < 140 mmHg was associated with a lower risk of all-cause mortality. Frailty may be a useful marker for determining whether antihypertensive treatment is effective for reducing all-cause mortality in older hypertensive patients.
